# The mitochondrial genome of *Telenomus remus* (Hymenoptera: Platygastridae)

**DOI:** 10.1080/23802359.2021.1884028

**Published:** 2021-03-15

**Authors:** Xiao-fei Li, Ze-kai Li, Jia-chen Zhu, Bo-ying Zheng, Pu Tang, Xue-xin Chen

**Affiliations:** a State Key Lab of Rice Biology, Zhejiang University, Hangzhou, China; b College of Agriculture and Biotechnology, Institute of Insect Sciences, Zhejiang University, Hangzhou, China; c Ministry of Agriculture Key Lab of Molecular Biology of Crop Pathogens and Insects, Zhejiang University, Hangzhou, China; d Zhejiang Provincial Key Laboratory of Biology of Crop Pathogens and Insects, Zhejiang University, Hangzhou, China

**Keywords:** Mitochondrial genome, *Telenomus remus*, Platygastridae, phylogeny

## Abstract

*Telenomus remus* Nixon, 1937 is an important parasitoid of lepidopterans. We sequenced the mitochondrial genome of *T. remus*, 15,500 bp in size, and possessed all 37 typical mitochondrial genes. A few tRNAs show gene arrangements compared with the ancestral gene order, mainly involving in the four tRNA clusters (*E-C*-*Y-Q-I-A*, *D-K*, *N-F-S1-R*, and *M-V*). The nucleotide sequences of 13 protein-coding genes of this sequence and another seven species from Platygastridae were used for phylogenetic analysis by MrBayes, with two species from Cynipoidea as an outgroup. The topology demonstrated that *T. remus* was most closely related to *Telenomus* sp.

*Telenomus remus* Nixon, 1937, an important egg parasitoid of various Lepidoptera species, belonging to Platygastridae of Hymenoptera. It originated from peninsular Malaysia, thought to be an excellent biological control agent and was introduced against *Spodoptera* spp. to various parts of the world (Nixon 1937; Bennett et al. 1985). In this study, we got the mt-genome of *T. remus* by the next-generation sequencing for the first time. It will facilitate a deeper understanding and exploitation of this species.

The sample of *T. remus* was collected from Ningbo (121°04′E, 30°15′N), Zhejiang Province, China in July 2019. The voucher specimen was stored in 100% ethanol and kept in the Parasitic Hymenoptera Collection of Institute of Insect Sciences, Zhejiang University (ZJUH_2020401, Pu Tang, ptang@zju.edu.cn). The whole genomic DNA was extracted from one female adult specimen using DNeasy tissue kit (Qiagen, Hilden, Germany). The library was constructed by VAHTS^TM^ Universal DNA Library Prep Kit for Illumina^®^ v3, and sequenced by Illumina HiSeq sequencer (150 bp pared-end). The reads were filtered by local BLAST with *E* value 1 × 10^−5^ referencing to all apocritan mitochondrial genomes dataset (downloaded from the GenBank database on 10 April 2020), and was assembled by Celera Assembler (Myers et al. 2000) and IDBA (Peng et al. 2012). The two assemblies were combined by GENEIOUS (Biomatters Ltd., San Diego, CA) to obtain a nonredundant set.

The mitochondrial genome of *T. remus* is 15,500 bp in length (GenBank accession MT906647), containing 13 protein-coding genes (PCGs), 22 transfer RNA (tRNA) genes, and two ribosomal RNA (rRNA) genes, but part of the control region (D-loop) failed to be sequenced or assembled. The overall base composition of the mitogenome was estimated to be A 44.6%, T 40.4%, C 8.8%, and G 6.3%, with a high A + T content of 85.0% and a little higher than that of *Telenomus* sp. (MF776884, 84.6%). All 13 PCGs of *T. remus* have the conventional ATN start codons for invertebrate mitochondrial PCGs (seven ATA, four ATT, and two ATG). All of the PCGs terminate with the stop codon TAA or TAG. The lengths of 16S rRNA and 12S rRNA in *T. remus* were 1289 and 770 bp, with the AT contents of 88.6% and 88.2%, respectively.

A few arrangements of tRNA genes compared with the ancestral gene order, where four main rearrangement events of tRNA clusters (*E-C*-*Y-Q-I-A*, *D-K*, *N-F-S1-R*, and *M-V*) were found in the sequenced region. All of these junctions are ‘hot spots’ where gene rearrangement occurs frequently in hymenopterans (Dowton and Austin 1999; Dowton et al. 2003; Wei et al. 2014). The arrangements are nearly identical to that of *Telenomus* sp., except for *E* moving from the cluster *N-E-F-S1-R* (*Telenomus* sp.) to *E-C-Y-Q-I-A* (*T. remus*).

To explore the phylogenetic position of *T. remus*, we used 13 PCGs of another seven mitochondrial genomes of Platygastridae and two species of Cynipoidea as an outgroup to perform phylogenetic analysis. The nucleotide sequences were aligned by using MAFFT v7.271. PartitionFinder v1.1.1 (Lanfear et al. 2012) was used to identify best partition scheme and substitution models, and the phylogenetic tree was constructed by MrBayes v3.2.549 (Ronquist et al. 2012). Phylogenetic analysis showed that *T. remus* was most closely related to *Telenomus* sp. Besides, *Telenomus* is closely related to *Trissolcus*, which was consistent with other results from the analyses based on morphological characters and several sequences before (Figure 1) (Kononova 2008; Taekul et al. 2014; Shen et al. 2019).

**Figure 1.**
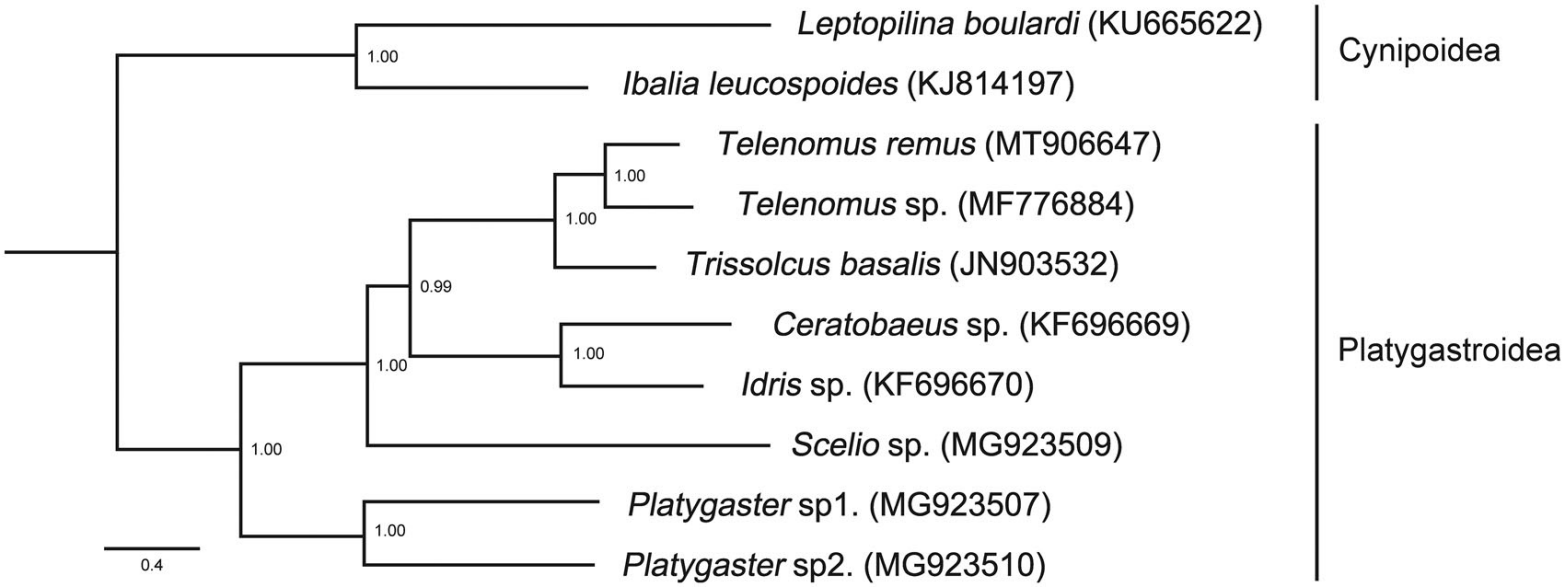
Phylogenetic tree of MrBayes using 13 PCGs in mitochondrial genomes of eight species in Platygastroidea with two species in Cynipoidea as outgroup. The numbers at the nodes are the Bayesian posterior probabilities.

## Data Availability

The data that support the findings of this study are openly available in GenBank of NCBI at https://www.ncbi.nlm.nih.gov. Mitochondrial genome data accession number MT906647. All high-throughput sequencing data files are available from the GenBank Sequence Read Archive (SRA) accession number PRJNA691080.
